# Gradients in the Number of Species at Reef-Seagrass Ecotones Explained by Gradients in Abundance

**DOI:** 10.1371/journal.pone.0020190

**Published:** 2011-05-24

**Authors:** Fernando Tuya, Mathew A. Vanderklift, Thomas Wernberg, Mads S. Thomsen

**Affiliations:** 1 Centro en Biodiversidad y Gestión Ambiental, Marine Sciences Faculty, Universidad de Las Palmas de Gran Canaria, Las Palmas, Canary Islands, Spain; 2 Centre for Marine Ecosystems Research, Edith Cowan University, Joondalup, Western Australia, Australia; 3 CSIRO Marine and Atmospheric Research, Wembley, Western Australia, Australia; 4 School of Plant Biology, Oceans Institute, University of Western Australia, Crawley, Western Australia, Australia; 5 Australian Institute of Marine Science, Crawley, Western Australia, Australia; University of Glamorgan, United Kingdom

## Abstract

Gradients in the composition and diversity (e.g. number of species) of faunal assemblages are common at ecotones between juxtaposed habitats. Patterns in the number of species, however, can be confounded by patterns in abundance of individuals, because more species tend to be found wherever there are more individuals. We tested whether proximity to reefs influenced patterns in the composition and diversity (‘species density’ = number of species per area and ‘species richness’ = number of species per number of individuals) of prosobranch gastropods in meadows of two seagrasses with different physiognomy: *Posidonia* and *Amphibolis*. A change in the species composition was observed from reef-seagrass edges towards the interiors of *Amphibolis*, but not in *Posidonia* meadows. Similarly, the abundance of gastropods and species density was higher at edges relative to interiors of *Amphibolis* meadows, but not in *Posidonia* meadows. However, species richness was not affected by proximity to reefs in either type of seagrass meadow. The higher number of species at the reef-*Amphibolis* edge was therefore a consequence of higher abundance, rather than species richness *per se*. These results suggest that patterns in the composition and diversity of fauna with proximity to adjacent habitats, and the underlying processes that they reflect, likely depend on the physiognomy of the habitat.

## Introduction

Habitats are connected within landscapes by multiple processes that influence the number of species (diversity) and the abundance of individuals at ecotones where habitats are juxtaposed. Gradients in diversity and abundance with proximity (or conversely, distance) to ecotones have been documented in terrestrial [1], [2] and marine ecosystems, both in temperate [3]–[6] and tropical regions [7].

Ecotones frequently contain a mixture of biota characteristic of the interiors of juxtaposed habitats [2]. Higher diversity at ecotones than in interiors of habitat patches might be due to this mixing [8]–[10]. However, patterns in diversity may also be confounded by patterns in abundance, because more species can be expected wherever there are more individuals. For example, ecotones can also host higher productivity or receive more propagules, processes which tend to increase the number of individuals, and this may also enhance diversity [11]. Conversely, ecotones can be subjected to elevated predation rates, which tend to decrease the number of individuals, and so reduce diversity [6], [12]. The counterbalance of these opposing forces can produce unexpected patterns in species diversity. Indeed, not all studies show higher species diversity at ecotones [13], [14]. Inconsistencies among studies can reflect real differences in ecology; for example, differences in plant physiognomy (i.e. the overall appearance of a plant, including its size and morphology) between habitats can affect patterns of diversity across ecotones [15]. However, inconsistencies might also simply reflect that different analytical methods have been used to evaluate diversity gradients at ecotones. It is important to distinguish between two different, but related, measures of species diversity: ‘species density’ (the number of species per unit area) *vs.* ‘species richness’ (the number of species per number of individuals) [16], [17].

Seagrass meadows are one of the most productive marine habitats, providing high-value ecosystem services [18]. Conservation of these valuable habitats is therefore important, particularly since seagrass meadows are in decline worldwide [19]. Seagrass meadows are widely distributed along temperate and tropical coasts [20], and are frequently interspersed as mosaics with other habitats, such as rocky reefs, coral reefs, mangroves, and unvegetated sediment [4], [7]. The biota of the meadows is typically different from the biota in adjacent habitats [6], [21], [22]. In some reef-seagrass landscapes, much of the production on reefs is exported to adjacent seagrass meadows [4], where it can be consumed by fauna inhabiting the seagrass [4], [23]. Proximity to reefs can also influence the intensity of ecological processes in seagrass meadows: for example, the supply of propagules can be higher close to reefs [21] which can increase the number of individuals, and foraging by predators can be more intense close to reefs [5], [7] which can decrease the number of individuals [10].

Here, we studied patterns in the composition and species diversity of prosobranch gastropod assemblages across reef-seagrass landscapes. We tested (i) whether differences in the composition of gastropod assemblages between reefs and seagrasses would increase from reef-seagrass ecotones to the interiors of seagrass meadows, and (ii) whether species density and species richness were higher at reef-seagrass ecotones than in the interiors of seagrass meadows. In particular, we sought to determine whether gradients in species richness existed independently from changes in composition and gradients in species density. We tested these predictions separately in meadows constituted by one of two species of seagrasses with contrasting physiognomy (*Posidonia* and *Amphibolis*) to (iii) determine whether differences in physiognomy can alter patterns in the composition and diversity of gastropod assemblages from reef-seagrass edges to interiors of seagrass meadows.

## Material and Methods

### Study area and survey design

The study was carried out on rocky reefs and adjacent seagrass meadows at two locations (∼250 km apart) in south-western Australia: Marmion (31°50′S) and Jurien Bay (30°18′S). In this area, the coast is characterized by sequences of limestone reefs parallel to the shore at distances ranging from <1 to 10 km offshore. Meadows of the seagrasses *Posidonia* spp. and *Amphibolis* spp. are interspersed among these reefs; this juxtaposition of reefs and seagrasses occurs along more than 1,500 km of coastline [4]. We selected 6 reefs, generally separated by >500 m, within each location: 3 adjacent to meadows dominated by *Posidonia* (mainly *P. sinuosa*, hereafter *Posidonia* meadow) and 3 adjacent to meadows dominated by *Amphibolis* (mainly *A. griffithii*, hereafter *Amphibolis* meadow). In the study area, reefs are predominantly covered by macroalgae, primarily the small (<1.5 m), canopy-forming, kelp *Ecklonia radiata*, and fucalean algae (mostly the genera *Sargassum* and *Scytothalia*). Patches of small (generally<25 cm) foliose red algae are interspersed between the stands of canopy-forming algae. All reefs had similar vertical relief: the height of reefs was, in all cases, 1–2 m higher than the surrounding seafloor. We took measurements at 5 distances relative to the reef-seagrass edge: on the reef itself (ca. 1–2 m from the reef-seagrass edge.), 0 m (first seagrass patch immediately adjacent to the reef), 10 m, 50 m, and >300 m away (first seagrass patch beyond the 300 m mark). Depths of the seagrass meadows ranged between 2–8 m; the direction of the gradient in proximity to reefs was typically oriented towards the shore. Although seagrass interiors generally were closer to the shore than the reef-seagrass edges, there were no depth gradients between seagrass edges and interiors, and *Posidonia* and *Amphibolis* meadows occurred at the same depths [6]. All surveyed reefs were <1 km from the adjacent mainland shore or the nearest island. Proximity to reefs does not significantly affect the physical architecture of each seagrass species, such as seagrass density, seagrass biomass and the biomass of epiphytic algae [6].

*Posidonia* and *Amphibolis* have different physiognomies; the strap-like leaves of *Posidonia* are uniform from base to tip, while *Amphibolis* has erect stems with small leaves arranged in clusters, forming a more complex canopy [24]. Shoot densities for *Posidonia* are generally higher than for *Amphibolis*, while above-ground biomass (including epiphytes) is typically higher for *Amphibolis* [25].

### Sampling

A SCUBA diver hand picked all above-ground vegetation, i.e. the habitat where gastropods live, within 5 replicate 25×25 cm quadrats haphazardly laid out at each distance. On reefs, collections were from haphazardly selected patches of red macroalgae, which in the study area typically host the same gastropod species as patches of brown macroalgae, but in much higher abundances [26]. In seagrass meadows, collections were from monospecific stands of either *Amphibolis* or *Posidonia*. Each sample was washed in fresh water, and passed through a 1 mm sieve. All gastropods retained were identified to the lowest possible taxonomic level. These procedures were repeated twice (i.e. two surveys): once each during austral summer-autumn of 2006 and 2007.

### Data analysis

For each combination of distance and seagrass, the total number of species was calculated by pooling all replicates at each distance across reefs and locations throughout the two surveys (n = 60). The total number of shared species between reefs and seagrass meadows at varying proximity from reefs was also calculated.

Canonical Analysis of Principal coordinates (CAP) [27] was used as an ordination procedure to visualize differences in gastropod composition between reefs and seagrass meadows at varying proximity to reefs. Incidence (presence/absence) data was used to remove the confounding effect of abundance, which would have caused a strong gradient regardless of composition. CAP provides a constrained ordination diagram that orients axes along the directions that best maximise differences among *a priori* groups. CAP was based on Jaccard dissimilarities calculated from presence/absence data. First, we conducted a Principal Coordinates Analysis (PCO); we then carried out the CAP based on the subset of the PCO axes at which additional axes did not add explanatory power. To facilitate visualization of patterns, variability among reefs and between replicates at each distance was averaged by using their corresponding centroids and data from the two surveys for each reef were pooled. Separate canonical analyses were done for *Posidonia* and *Amphibolis* meadows to maximize the allocation success of points in the ordination space [27]. The CAP routine calculated misclassification errors for centroids at distances away from reefs using the ‘Leave-one-out Allocation success’ (LoA). Comparison of known groups with allocated groups provided misclassification errors [27]. To test for differences in the composition of gastropod assemblages with varying proximity from reefs for each seagrass and survey, we partitioned the variability in assemblage composition via a Permutational Multivariate Analysis of Variance (PERMANOVA) [28] performed on Jaccard dissimilarities calculated from presence/absence data. The model included: ‘Locations’ (random factor), ‘Reefs’ (random factor nested within locations) and ‘Distances’ away from reefs (fixed factor, orthogonal to both previous factors). Separate analyses were performed for each seagrass species and survey. Pair-wise comparisons between each pair of distances were executed (via 4999 permutations) when significant differences were found for the factor ‘distances’. The same model, but in a univariate context, tested for differences in the total abundance of gastropods with varying proximity from reefs for each seagrass and survey. Abundance data was Ln(x+1) transformed to achieve homogeneous variances, and permutation-based pairwise comparisons (via 4999 permutations) tested for differences between each pair of distances when significant differences were found for the factor ‘distances’. All multivariate procedures were carried out via the PRIMER 6.0 & PERMANOVA+ statistical package.

For each combination of distance and seagrass, species density and species richness were estimated by rarefying the data using sample- and individual-based rarefaction methods, respectively [17]. Comparisons of species density were carried out for the total number of replicates at each distance (n = 60 for collections at each distance), while comparisons of species richness were per 170 individuals (the lowest number per collection) [29]. Confidence intervals (95%) were calculated with a bootstrap procedure [29], [30] to allow for meaningful comparisons of species density and species richness among distances for each seagrass species; the overlapping of 95% confidence intervals was then taken into account to test for significant results. All computations were carried out through the *Estimate*S software [31]. For these analyses, data were pooled across locations and reefs within each location (random spatial variation), throughout the two surveys (random temporal variation).

## Results

**Figure 1:**
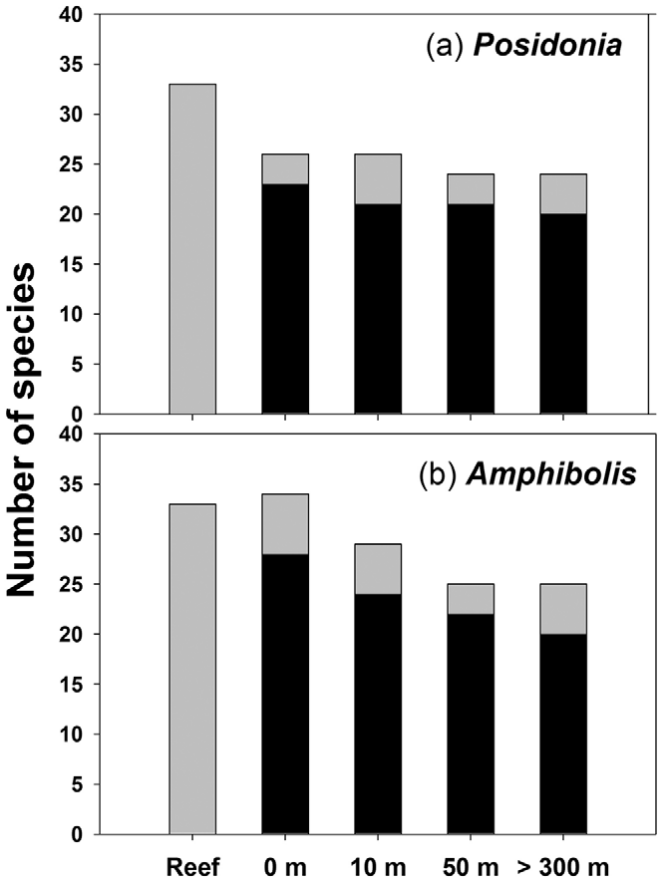
Total number of gastropod species on reefs and seagrass meadows with varying proximity to reefs. (a) *Posidonia*; (b) *Amphibolis*. The number of shared taxa between reefs and seagrass meadows at varying proximity from reefs is nested (i.e. black bar) inside each bar.

**Figure 2:**
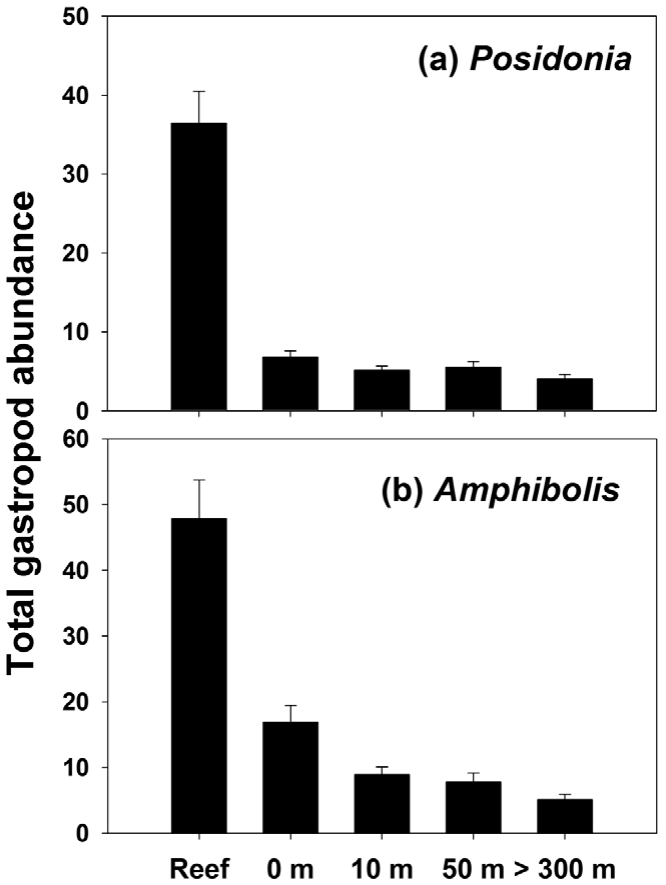
Total abundance (mean + SE, n = 60) of gastropods on reefs and seagrass meadows with varying proximity to reefs. (a) *Posidonia*; (b) *Amphibolis*.

A total of 15,368 individuals and 43 species of gastropods were counted on reefs and adjacent seagrass meadows. Five species were unique to reefs, while four species were unique to seagrass meadows. The total number of gastropod species generally declined monotonically from reef edges to the interiors of seagrass meadows (Fig. 1). The total number of shared taxa between reefs and seagrass meadows also declined with increasing distance from reefs, though this decline depended on the dominant seagrass species: reefs and reef-seagrass edges shared a larger number of taxa in *Amphibolis* meadows (Fig. 1b). The total abundance of gastropods (Fig. 2) was higher on reefs than in seagrass meadows (Table 1; P<0.05 for “Distances” in all cases). Total abundances were higher at reef-seagrass edges (0 m) than in *Amphibolis*-seagrass interiors (>300 m) (Fig. 2, P<0.05, pairwise comparisons), but not in *Posidonia*-seagrass interiors (Fig. 2, P>0.05, pairwise comparisons).

**Table 1:** Uni and multivariate-ANOVA testing for differences in total gastropod abundance and gastropod assemblage composition, respectively.

	*Posidonia*							*Amphibolis*					
		Composition			Total abundance			Composition			Total abundance		
Survey 1	df	MS	F	P	MS	F	P	MS	F	P	MS	F	P
Loc = Locations	1	41785.07	2	0.1	63.58	8.48	0.04	39852.47	3.67	0.09	7.47	1.74	0.25
Reefs (Loc)	4	20811.05	11.08	0.001	7.5	24.63	0.001	11155.97	5.76	0.001	4.3	8.87	0.001
Dis = Distances	4	13037.97	1.71	0.091	18.79	9.94	0.023	18807.79	4.15	0.012	17.15	17.03	0.008
Loc×Dis	4	7596.81	1.81	0.023	1.89	0.76	0.565	4525.99	1.19	0.27	1.01	2.09	0.13
Reef (Loc)×Dis	16	4194.07	2.23	0.001	2.48	8.14	0.001	3799.16	1.96	0.001	0.48	1	0.465
Residual	120	1878.11			0.3			1935.56			0.48		

Survey 2													
Loc	1	43582.92	2.12	0.09	23.06	5.57	0.077	38772.09	1.71	0.15	8.42	0.95	0.383
Reefs (Loc)	4	20478.04	10.4	0.01	4.14	11.35	0.001	22642.53	10.29	0.01	8.83	23.83	0.001
Dis	4	13422.51	1.52	0.1	22.22	13.46	0.013	18983.21	1.94	0.07	15.52	16.66	0.009
Loc×Dis	4	8808.16	1.63	0.04	1.65	1.68	0.203	9766.82	1.49	0.07	0.93	0.56	0.691
Reef (Loc)×Dis	16	5398.65	2.74	0.001	0.98	2.69	0.001	6543.33	2.97	0.001	1.65	4.45	0.001
Residual	120	1968.65			0.36			2198.76			0.37		

Results for differences in total gastropod abundance and composition between locations, reefs within locations, and distances away from reefs for each seagrass species and survey.

**Figure 3:**
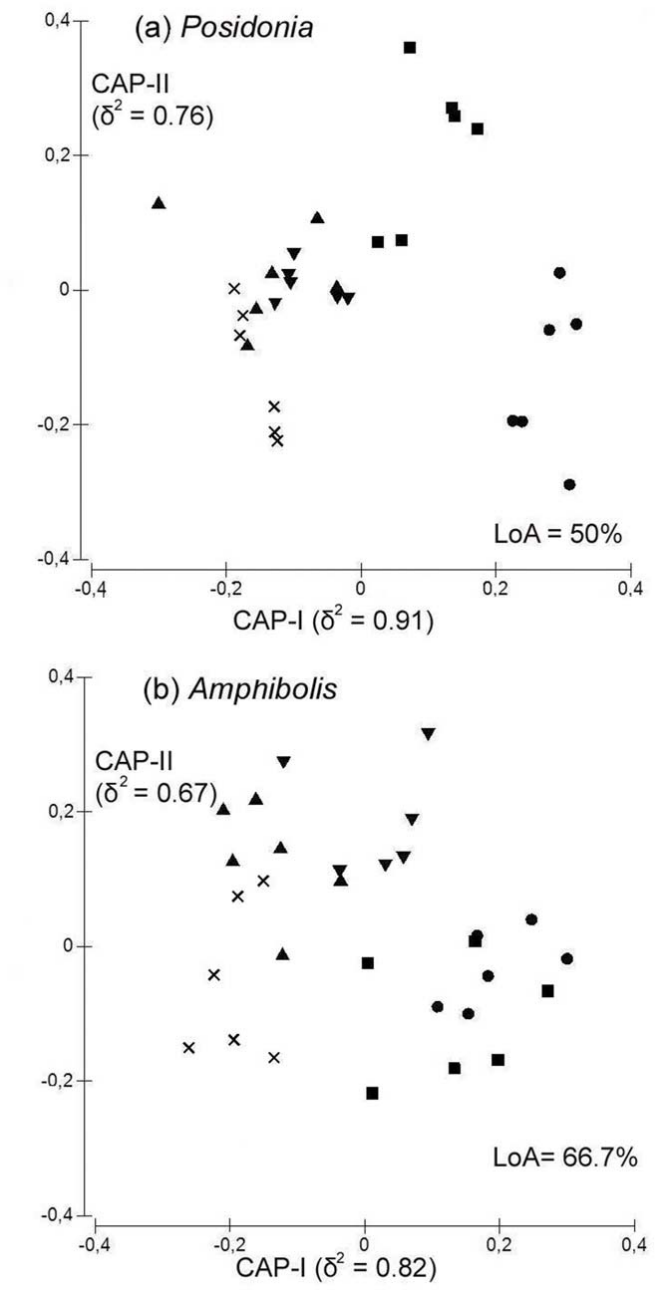
Canonical ordination plots (CAP) of gastropod assemblage composition on reefs and seagrass meadows with varying proximity to reefs. (a) *Posidonia*; (b) *Amphibolis*. ×: Reef, ▴: 0 m, ▾: 10 m ▪: 50 m, •: >300 m.

A change in the composition of gastropod species on seagrass meadows with distance away from reefs was evident in the CAP plots (Fig. 3). Gastropod assemblages from reef-seagrass edges (0 m) and immediately adjacent seagrasses (10 m away from reefs) tended to cluster closer to those from reefs than to assemblages in the interiors of seagrass meadows (50 and >300 m away from reefs). This change in species composition with proximity from reefs, however, was more accentuated on *Amphibolis* (Table 1; P≤0.07 for “Distance” in both surveys) than on *Posidonia* meadows (Table 1; 0.07<P<0.10 for “Distance” in both surveys). Indeed, the ‘Leave-one-out Allocation’ success (LoA, i.e. the percentage of points correctly allocated into each distance, Fig. 3) was higher for *Amphibolis* (66.7%) than *Posidonia* meadows (50%), which indicates a higher misclassification for centroids of distances away from reefs for *Posidonia* meadows than *Amphibolis* meadows. Differences in assemblage composition among distances away from reefs, however, varied from reef to reef (Table 1; P<0.01, ‘Reef (Loc)×Dis)’ in both surveys). Pairwise comparisons of species composition among distances away from each reef (Table 2) revealed that the species composition of gastropod assemblages differed significantly between reefs and reef-seagrass ecotones (0 m) at only 6 and 4 reefs (from a total of 12, Table 2) in *Posidonia* and *Amphibolis* meadows, respectively. Significant differences in the composition of gastropod assemblages between reefs and seagrass meadow interiors (>300 m away from reefs) were observed, however, at all reefs (Table 1), in both *Posidonia* and *Amphibolis* meadows.

**Table 2:** Pairwise comparisons testing for differences in the composition of gastropod assemblages among each reef and adjacent seagrass meadows at varying proximity from each reef.

	Survey 1		Survey 2	
	*Posidonia*	*Amphibolis*	*Posidonia*	*Amphibolis*
Reef vs. 0 m	3 (50%)	2 (33.33%)	3 (50%)	2 (33.33%)
Reef vs. 10 m	5 (83.34%)	3 (50%)	4 (66.66%)	3 (50%)
Reef vs. 50 m	4 (66.66%)	6 (100%)	4 (66.66%)	6 (100%)
Reef vs. >300 m	6 (100%)	6 (100%)	6 (100%)	6 (100%)
0 m vs. 10 m	0	0	0	0
0 m vs. 50 m	1 (16.66%)	3 (50%)	1 (16.66%)	2 (33.33%)
0 m vs. >300 m	5 (83.34%)	5 (83.34%)	5 (83.34%)	6 (100%)
10 m vs. 50 m	0	2 (33.33%)	0	2 (33.33%)
10 m vs. >300 m	2 (33.33%)	5 (83.34%)	2 (33.33%)	4 (66.66%)
50 m vs. >300 m	0	2 (33.33%)	0	1(16.66%)

Results are presented for each seagrass species and survey (survey 1 = austral summer-autumn 2006, survey 2 = austral summer-autumn 2007). The number and percentage of reefs (from a total of 6 reefs per seagrass and survey) where we detected a significant difference (P<0.01) is indicated.

**Figure 4:**
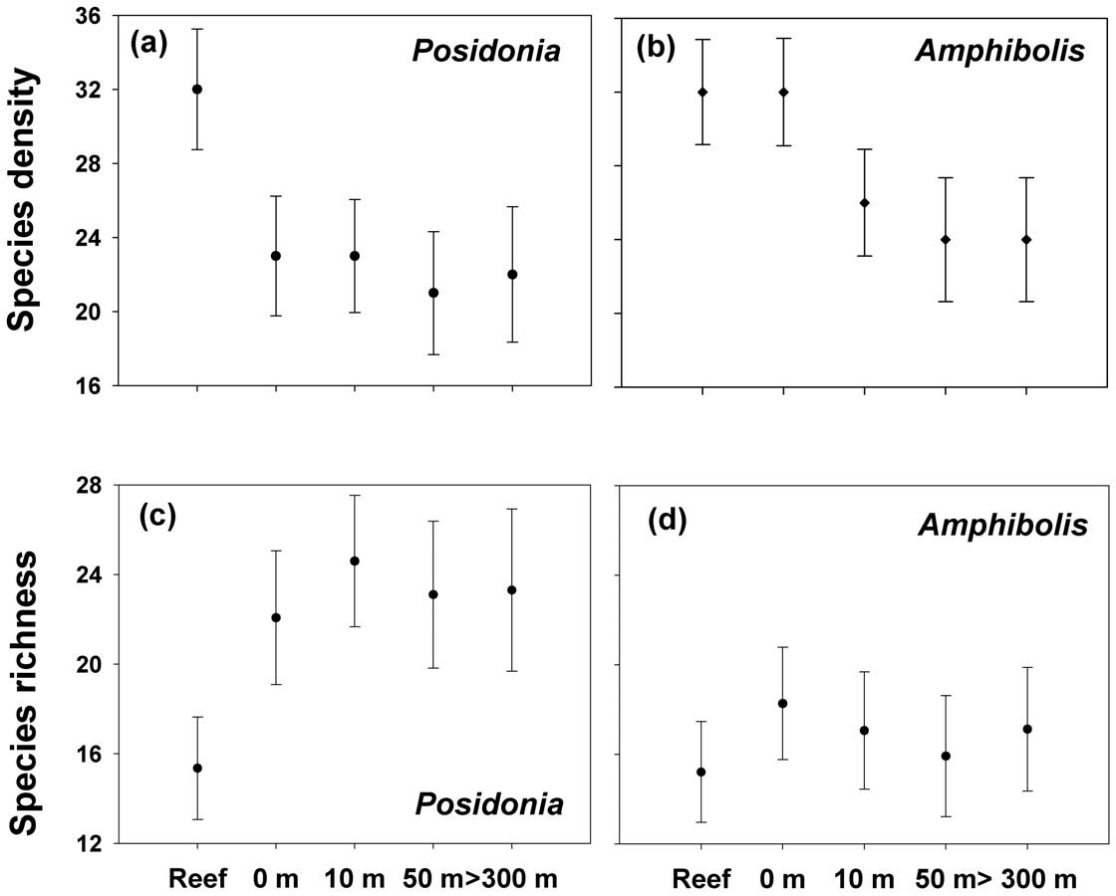
Patterns of species density (species/area; a, b) and species richness (species/individuals; c, d) of gastropods on reefs and seagrass meadows with varying proximity to reefs. Values are means ±95% confidence intervals.

The species density of gastropods was higher on reefs than in *Posidonia* meadows at all distances away from reefs, and higher on reefs than *Amphibolis* meadows at all distances beyond 0 m (Figs. 4a and 4b, i.e. 95% confidence intervals do not overlap). The species density of gastropods was higher at the reef-seagrass edge (0 m) relative to *Amphibolis* meadows at 10, 50 and >300 m away from reefs (Fig. 4b). Conversely, rarefied species richness was higher in *Posidonia* meadows relative to reefs, irrespective of proximity from reefs (Fig. 4c). In *Amphibolis* meadows, no differences in rarefied species richness between reefs and seagrasses were observed at any distance (Fig. 4d).

## Discussion

The similarity in the composition of assemblages inhabiting adjacent habitats tends to decrease with increasing distance from habitat edges. This pattern has been observed for plant and animal assemblages inhabiting both terrestrial [32], [33] and marine ecosystems [3], [21]. Our study confirms this pattern for seagrass-associated gastropods where seagrass meadows are interspersed with reefs, in particular where reefs are covered by red algal patches. We found that the total abundance and number of species, and species composition, of gastropods inhabiting seagrass meadows tended to decline with distance from reefs. However, we found that this pattern reflected patterns in species density, not patterns of species richness.

In general, the number of species (species diversity) declines with distance from edges where two habitats are juxtaposed [9], [34]. The most commonly invoked explanation is that there is a ‘mixing of biotas’ associated with each habitat at edges, giving rise to an area of overlap with a larger overall species pool [9], [10]. While this is a common pattern for species diversity between edges and interiors, it is not universal: several studies have found either no ‘edge effect’ or even increases in the number of species with distance from edges [13], [14], [33]. In our study, differences in species density between reef-seagrass edges and the interiors of seagrass meadows depended on the seagrass species. The higher number of species at the reef-*Amphibolis* edge (0 m) was therefore a consequence of a higher species density – that is more individuals -, not higher species richness *per se*.

Higher abundances of gastropods have previously been found in *Amphibolis* relative to *Posidonia* meadows in the study area [6], [25], [35]. Such differences have been attributed to differences in the physical structure and longevity of above-ground parts between these two seagrass species [24], [25]. *Amphibolis* forms a denser and more complex canopy with longer-lived stems, and also hosts greater biomass and more species of large erect epiphytes [36]. It is thus plausible that the number of microhabitats per area is larger in *Amphibolis* than *Posidonia* seagrass meadows, which might explain the higher abundance of gastropods in *Amphibolis* meadows. This was evidenced by the fact that the number of shared taxa between reefs and adjacent seagrasses (i.e. 0 and 10 m away from reefs) was larger in *Amphibolis* than *Posidonia* meadows.

Dispersal of fauna between adjacent habitats may occur via physical vectors; e.g. wind patterns can favour the spillover of insects across crop and non-crop ecotones in terrestrial ecosystems [37]. A high degree of connectivity between gastropods inhabiting reefs and immediately adjacent seagrass meadows is likely to be due to the high dispersal ability of most gastropods [38]. As juveniles or adults, gastropods may disperse by crawling or drifting as a result of water motion [39]. Dislodgement of gastropods is widespread during periods of high surge (i.e. big swells) on intertidal and shallow subtidal reefs [40]. For example, some gastropod populations (e.g. *Pyrene bidentata*) inhabiting seagrasses adjacent to reefs might be maintained by immigration from abundant reef populations [6], reflecting that proximity to sources of new individuals is an important influence on patterns of composition and abundance [41]. In addition, gradients in propagule release, larval dispersion, and recruitment with proximity to reefs may also contribute to explain differences in the composition and abundance between the edges and interiors of seagrass meadows [21].

In summary, this study has demonstrated a change in the composition of assemblages, from the reef-seagrass edge towards the interiors of seagrass meadows. Species density was higher at reef-seagrass edges than meadow interiors for *Amphibolis* meadows, but not *Posidonia* meadows. The patterns for *Amphibolis* meadows are most consistent with a higher number of species as a result of more individuals rather than species richness *per se*. These results suggest, therefore, that a change in the composition and diversity of fauna with proximity to adjacent habitats likely depend on the physiognomy of the habitat.

## References

[1] Lahti DC (2001). The “edge effect on nest predation” hypothesis after twenty years.. Biol Conser.

[2] Rand TA, Tylianakis JM, Tsacharntke T (2006). Spillover edge effects: the dispersal of agriculturally subsidized insect natural enemies into adjacent natural habitats.. Ecol Lett.

[3] Barros F, Underwood AJ, Lindegarth M (2001). The influence of rocky reefs on the structure of benthic macrofauna in nearby soft-sediments.. Estuar Coast Shelf Sci.

[4] Wernberg T, Vanderklift MA, How J, Lavery PS (2006). Export of detached macroalgae from reefs to adjacent seagrass beds.. Oecologia.

[5] Vanderklift MA, How J, Wernberg T, MacArthur LD, Heck KL (2007). Proximity to reef influences density of small predatory fishes, while type of seagrass influences intensity of their predation on crabs.. Mar Ecol Prog Ser.

[6] Tuya F, Vanderklift MA, Hyndes GA, Wernberg T, Thomsen MS (2010). Proximity to reefs affects the balance between positive and negative effects on seagrass fauna.. Mar Ecol Prog Ser.

[7] Valentine JF, Heck KL, Blackmon D, Goecker ME, Christian J (2008). Impacts of exploited species on food web interactions along the coral reef-seagrass interface: a comparison using fished and no-take zones in the Florida Keys National Marine Sanctuary.. Ecol Appl.

[8] Odum EP (1971). Fundamentals of ecology..

[9] Magura T (2002). Carabids and forest edge: spatial pattern and edge effect.. Forest Ecol Manag.

[10] Ries L, Fletcher RJ, Battin J, Sisk TD (2004). Ecological responses to habitat edges: mechanisms, models, and variability explained.. Ann Rev Ecol Syst.

[11] Talley DM, Huxel GR, Holyoak M, Crooks KR, Sanyajan M (2006). Connectivity at the land-water interface.. Connectivity Conservation.

[12] Kark S, van Rensburg BJ (2006). Ecotones: Marginal or central areas of transition?. Israel J Ecol Evol.

[13] Risser PG (1995). The status of the science examining ecotones.. BioScience.

[14] Baker J, French K, Whelan RJ (2002). The edge effect and ecotonal species: bird communities across a natural edge in southeastern Australia.. Ecology.

[15] Walker S, Wilson JB, Steel JB, Rapson GL, Smith B (2003). Properties of ecotones: Evidence from five ecotones objectively determined from a coastal vegetation gradient.. J Veg Sci.

[16] Hulbert SH (1971). The non-concept of species diversity: a critique and alternative parameters.. Ecology.

[17] Gotelli NJ, Colwell RK (2001). Quantifying biodiversity: procedures and pitfalls in the measurement and comparison of species richness.. Ecol Lett.

[18] Unsworth RKF, Cullen LC (2010). Recognising the necessity for Indo-Pacific seagrass conservation.. Cons Letters.

[19] Waycott M, Duarte C, Carruthers TJB, Orth RJ, Dennison WC (2009). Accelerating loss of seagrasses across the globe threatens coastal ecosystems.. Proc Natl Acad Sci USA.

[20] Hemminga MA, Duarte CM (2000). Seagrass Ecology..

[21] Van Elven BR, Lavery PS, Kendrick GA (2004). Reefs as contributors to diversity of epiphytic macroalgae assemblages in seagrass meadows.. Mar Ecol Prog Ser.

[22] Bostrom C, Jackson EL, Simenstad CA (2006). Seagrass landscapes and their effects on associated fauna: A review.. Estuar Coast Shelf Sci.

[23] Doropoulos C, Hyndes GA, Lavery PS, Tuya F (2009). Dietary preferences of two seagrass inhabiting gastropods: allochthonous vs. autochthonous resources.. Estuar Coast Shelf Sci.

[24] Edgar GJ (1992). The influence of plant structure on the species richness, biomass and secondary production of macrofaunal assemblages associated with Western Australian seagrass beds.. J Exp Mar Biol Ecol.

[25] Jernakoff P, Nielsen J (1998). Plant–animal associations in two species of seagrasses in Western Australia.. Aquat Bot.

[26] Tuya F, Wernberg T, Thomsen MS (2008). The spatial arrangement of reefs alters the ecological patterns of fauna between interspersed habitats.. Estuar Coast Shelf Sci.

[27] Anderson MJ, Willis TJ (2003). Canonical analysis of principal coordinates: a useful method of constrained ordination for ecology.. Ecology.

[28] Anderson MJ (2001). Permutation tests for univariate and multivariate analysis of variance and regression.. Canadi J Fish Aquat Sci.

[29] Collins MD, Simberloff D (2008). Rarefaction and non-random spatial dispersion patterns.. Environ Ecol Stat.

[30] Colwell RK, Mao CX, Chang J (2004). Interpolating, extrapolating, and comparing incidence-based species accumulation curves.. Ecology.

[31] Colwell RK (2000). EstimateS: Statistical Estimation of Species Richness and Shared Species from Samples (Software and User's Guide), Version 6.. http://viceroy.eeb.uconn.edu/estimates.

[32] Dangerfield JM, Pik AJ, Britton D, Holmes A, Gillings M (2003). Patterns of invertebrate biodiversity across a natural edge.. Aust Ecol.

[33] Dutoit T, Buisson E, Gerbaud E, Roche P, Tatoni T (2007). The status of transitions between cultivated fields and their boundaries: ecotones, ecoclines or edge effects?. Acta Oecologica.

[34] Denys C, Tscharntke T (2002). Plant-insect communities and predator-prey ratios in field margin strips, adjacent crop fields, and fallows.. Oecologia.

[35] Sergeev VN, Clarke SM, Shepherd SA (1988). Motile macroepifauna of the seagrasses, *Amphibolis* and *Posidonia*, and unvegetated sandy substrata in Holdfast Bay, South Australia.. Transactions of the Royal Society of South Australia.

[36] Lavery PS, Vanderklift MA (2002). A comparison of spatial and temporal patterns in epiphytic macroalgal assemblages of the seagrasses *Amphibolis griffithii* and *Posidonia coriacea*.. Mar Ecol Prog Ser.

[37] Tscharntke T, Rand TA, Bianchi FJJA (2005). The landscape context of trophic interactions: insect spillover across the crop non-crop interface.. Ann Zool Fennici.

[38] Tuya F, Wernberg T, Thomsen MS (2009). Colonization of gastropods on subtidal reefs depends on density in adjacent habitats, not disturbance regime or latitude.. J Mollus Stu.

[39] Jorgensen NM, Christie H (2003). Diurnal, horizontal and vertical dispersal of kelp-associated fauna.. Hydrobiologia.

[40] Miller LP, O'Donnell MJ, Mach KJ (2007). Dislodge but not dead: survivorship of a high intertidal snail following wave dislodgement.. J Mar Biol Assoc UK.

[41] Witman JD, Dayton P, Bertness MD, Gaines SD, Hay ME (2001). Rocky subtidal communities.. Marine Community Ecology.

